# Predicting range shifts of *Davidia involucrata* Ball. under future climate change

**DOI:** 10.1002/ece3.8023

**Published:** 2021-08-11

**Authors:** Teng Long, Junfeng Tang, Nicholas W. Pilfold, Xuzhe Zhao, Tingfa Dong

**Affiliations:** ^1^ Key Laboratory of Southwest China Wildlife Resources Conservation (Ministry of Education) China West Normal University Nanchong China; ^2^ Conservation Science and Wildlife Health San Diego Zoo Wildlife Alliance Escondido CA USA

**Keywords:** climate change, *Davidia involucrate*, dove tree, ensemble species distribution models (SDMs), habitat suitability, range shifts

## Abstract

Understanding and predicting how species will respond to climate change is crucial for biodiversity conservation. Here, we assessed future climate change impacts on the distribution of a rare and endangered plant species, *Davidia involucrate* in China, using the most recent global circulation models developed in the sixth Assessment Report of the Intergovernmental Panel on Climate Change (IPCC6). We assessed the potential range shifts in this species by using an ensemble of species distribution models (SDMs). The ensemble SDMs exhibited high predictive ability and suggested that the temperature annual range, annual mean temperature, and precipitation of the driest month are the most influential predictors in shaping distribution patterns of this species. The projections of the ensemble SDMs also suggested that *D. involucrate* is very vulnerable to future climate change, with at least one‐third of its suitable range expected to be lost in all future climate change scenarios and will shift to the northward of high‐latitude regions. Similarly, at least one‐fifth of the overlap area of the current nature reserve networks and projected suitable habitat is also expected to be lost. These findings suggest that it is of great importance to ensure that adaptive conservation management strategies are in place to mitigate the impacts of climate change on *D. involucrate*.

## Graphical abstract

*Davidia involucrate* is very vulnerable to future climate change, with at least one third of its suitable range expected to be lost in all future climate change scenarios and will shift to the northward of high‐latitude regions

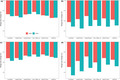

## 1 INTRODUCTION

The recent global warming due to the increase of greenhouse gases caused by human activities is driving global species redistributions (Hampe & Petit, 2005; Jackson & Sax, 2010; Pearson & Dawson, 2003). In response to global warming, many species have attempted to keep pace with climate change by adjusting their phenology and physiology to match new climatic conditions (Walther et al., 2002), or shifting their distributions toward higher altitudes or latitudes to track suitable habitats (Chen et al., 2011; Hampe & Petit, 2005; Jarzyna et al., 2021; Jump & Penuelas, 2005; Tehrani et al., 2020; Walther et al., 2002). Unfortunately, those species that failed to shift their distribution lost a substantial proportion of their suitable habitats or have even became extinct globally (Flagmeier et al., 2014; Sproull et al., 2015). Furthermore, this situation may worsen under future climate change (Thomas et al., 2014; Warren et al., 2013). Thus, in order to mitigate the negative effects of climate change on species, conservation strategies should be refined by modeling species distributions to identify to what extent they could be influenced by future climate change (Ramirez‐Villegas et al., 2014; Thuiller et al., 2008).

In the past two decades, species distribution models (SDMs) have been widely used to assess the impacts of future climate change on species distributions and guide conservation planning (Kujala et al., 2013; Maggini et al., 2014; Wiens et al., 2009). However, SDMs may suffer from a lack of precision and portability due to the variation in covariate selections (Zhang & Zhang, 2012), type of SDM used (Hartley et al., 2006; Pearson et al., 2006; Thuiller et al., 2009; Wenger et al., 2013), and climate projections arising from different global circulation models (GCMs) and CO_2_ emission scenarios (Barry & Elith, 2006; Wenger et al., 2013), which can yield misleading or inconsistent outcomes, posing challenges for decision‐making (Elith et al., 2006). Ensemble modeling approaches, which combined a series of SDMs, can produce consensus projections that may outperform single SDMs (Araújo & New, 2007; Marmion et al., 2009) and reduce the predictive uncertainty of single algorithm by combining their predictions (Tehrani et al., 2021; Thuiller et al., 2014). By using ensemble modeling approaches, more robust projections can be produced with reasonable interpretation (Araújo & Guisan, 2006). Consequently, these approaches have been widely used to estimate the distributions of species under future climate change scenarios for plants (Forester et al., 2013), amphibians (Zhang, Dong, et al., 2020; Zhang, Mammola, et al., 2020), insects (Marshall et al., 2018), and mammals (Ahmad et al., 2020; Yen et al., 2011).

*Davidia involucrata* Baill., commonly known as dove tree or handkerchief tree, is a rare and endangered species listed in the China Plant Red Data Book under first‐grade state protection (Liu et al., 2019). It is also a Tertiary relict plant endemic to China (Fu & Jin, 1992), currently ranges approximately from 98–110°E, 26–32°N in southwestern and south‐central China, including Yunnan, Guizhou, Sichuan, southern Shaanxi, southern Gansu, Chongqing, Hubei, and Hunan Provinces (Li, 1954; Liu et al., 2019; Takhtajan, 1980; Tang et al., 2017). Its populations are often found in subtropical evergreen broad‐leaved forests or in mixed forests of temperate deciduous broad‐leaved trees at altitudes of between1100 and 2,600 m (He et al., 2004). Owing to the highly strict ecotope and recruitment limitation (i.e., low reproduction rate and limited dispersal ability), the population age structure of *D. involucrate* is declining (Wang Yu‐sheng et al., 2019). In addition, the increasing intensity of human activities (e.g., logging) has led to a sharp decrease of its remaining habitats (Wang Yu‐sheng et al., 2019). Despite its threatened status, few studies have explored the vulnerability of *D. involucrate* to climate change (Tang et al., 2017; Wang Yu‐sheng et al., 2019). By using ecological niche models (ENMs) (Boria et al., 2014; Peterson, 2006), Tang et al. (2017) projected the potential suitable habitats of this species under past, current, and future climatic conditions. In their work, the obsolete CMIP5 climate models were used to simulate future climate conditions (Tang et al., 2017). However, many studies have shown that the most recent CMIP6 climate models perform better in the simulation of future climate conditions compared to the CMIP5 climate models (Fan et al., 2020; Xin et al., 2020). Therefore, a rigorous analysis combined ensemble modeling approaches and the CMIP6 climate models investigating potential impacts of future climate change on the distribution of *D. involucrate* is of great urgency and significance.

In this study, we aim to (a) assess the vulnerability of *D. involucrate* to climate change (i.e., whether the suitable habitats of D. involucrate will suffer great lost under future climate change), and (b) evaluate the conservation effectiveness of current nature reserve networks in protecting *D. involucrate* under climate change. To this end, we compiled a large dataset on spatially explicit species presence records of the *D. involucrate* and environmental data (bioclimatic variables) covering China and subsequently used ensemble modeling approaches to project the potential suitable habitats of *D. involucrate* under current and future climatic conditions. According to our knowledge, our study is one of the first studies to investigate how *D. involucrate* will response to future climate change by using ensemble modeling approaches and the most recent CMIP6 global circulation models.

## 2 MATERIAL AND METHODS

### 2.1 Study area and species occurrence data

**FIGURE 1:**
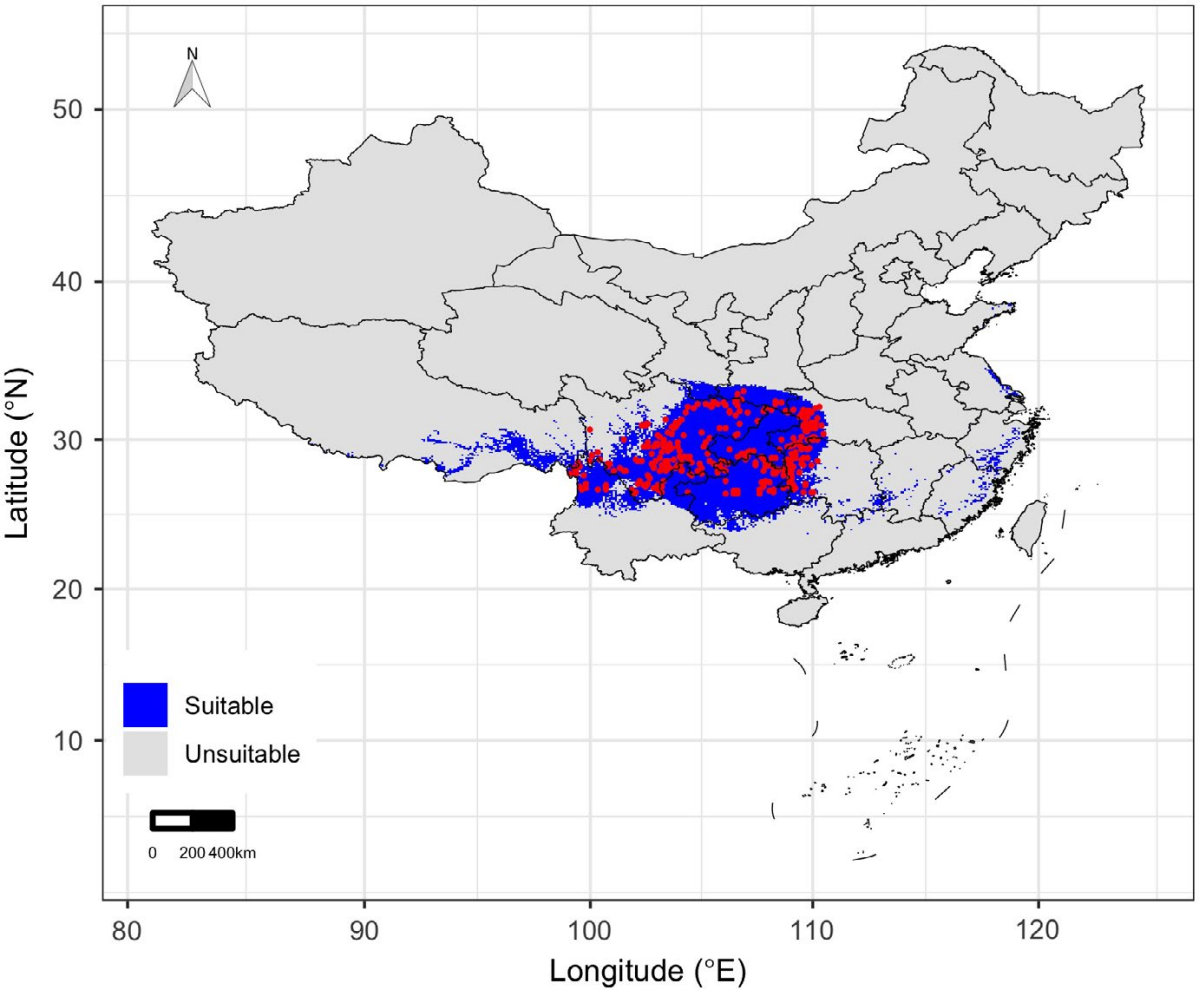
Potential suitable (blue) and unsuitable (gray) habitats suitability of *Davidia involucrata* Baill. under current climatic conditions in China. Red points represent occurrence records of *D*. *involucrata*

According to previous studies on the suitable habitat of *D. involucrate*, we chose the whole China as the study area (Figure 1). As a Tertiary relict plant endemic to China, *D. involucrate* has been widely studied and long‐term national observation records are available in China (Tang et al., 2017; Wang Yu‐sheng et al., 2019), which makes China immensely suitable for assessing the potential effects of climate change on the geographical distribution of *D. involucrate*. Here, we assembled an database of occurrence records of *D. involucrate* in China from multiple data sources, including Chinese Virtual Herbarium (CVH, http://www.cvh.ac.cn), National Specimen Information Infrastructure (NSII, www.nsii.org.cn), Global Biodiversity Information Facility (GBIF, https://www.gbif.org), and other publications (e.g., Wang Yu‐sheng et al., 2019). These occurrence records were mainly collected from field surveys, which were carried out between the years 1971 and 2017. Overall, 793 occurrence records of *D. involucrate* were collected. These occurrence records were mainly distributed in 198 nature reserves (the Ministry of Environmental Protection, http://www.mep.gov.cn/stbh/zrbhq/qgzrbhqml/). As our climate data were from the years 1979–2013 (see below), we excluded the occurrence records that were not collected from this time period. Furthermore, to reduce potential errors in species' geographic locations of occurrence data, we only include species occurrence records with geographic locations. Finally, to ensure that all occurrence records were in the species' native geographic locations, we also excluded the species occurrence records collected in manual intervention areas, such as parks and experimental forests. From this process, we had 337 occurrence records available to use (Figure 1).

In order to reduce the high uncertainty in geographic coordinates of the occurrence records and minimize the sampling bias effect in the occurrence records dataset, all the 337 occurrence records were compiled at a spatial resolution of 10 × 10 km grids cell. After removing duplicate records within each gird cell, we obtained 324 presence records to model ecological niches for *D. involucrate*.

### 2.2 Climatic variable

19 bioclimatic variables (BIO1‐BIO19; Appendix S1) for the time period 1979–2013 were obtained from CHELSA (http://chelsa‐climate.org; Booth et al., 2014; Busby, 1988; Karger et al., 2017), with a spatial resolution of 30 arc‐seconds (~1 km). The future 19 bioclimatic variables with a 2.5 arc‐minutes resolution for two time periods, 2050s (2041–2060) and 2070s (2061–2080), under two representative concentration pathways (RCPs) scenarios, RCP2.6 and RCP8.5, from six widely used global circulation models (GCMs): CNRM‐CM6‐1, CNRM‐ESM2‐1, CanESM5, IPSL‐CM6A‐LR, MIROC‐ES2L, and MIROC6, were extracted directly from the WorldClim Version 2.1 dataset (Fick & Hijmans, 2017). For each time period, each GCM and for each RCP scenarios, we projected all maps of current and future climate variables onto the same 10‐km equal area grid as used for fitting the distribution models, using a bilinear interpolation.

The 19 climate variables used in this study are usually strongly correlated (Marshall et al., 2018). To minimize multicollinearity among variables, we used Pearson's correlations and variance inflation factors (VIFs) to exclude highly correlated variables. Variables with a Pearson correlation >0.70 were considered highly correlated (Dormann et al., 2013), and a VIF >5 was used as a signal that a model had collinearity issues (Rogerson, 2010). Finally, six climate variables were selected for modeling species distributions, including annual mean temperature (BIO1), isothermality (BIO3), temperature annual range (BIO7), precipitation of the driest month (BIO14), precipitation seasonality (BIO15), and precipitation of the warmest quarter (BIO18) (Appendix S2).

### 2.3 Species distribution modeling

An ensemble of species distribution models (Araújo & Guisan, 2006) was used to model potential suitable habitat for *D. involucrata* using the biomod2 package in the R platform (v. 4.0.4; http://cran.r‐project.org). We chose the ensemble modeling approach because of its ability to create a consensus of the predictions of multiple algorithms and reduce the predictive uncertainty of single algorithm (Kanagaraj et al., 2019; Thuiller et al., 2014; Zhang, Dong, et al., 2020; Zhang, Mammola, et al., 2020). Ten algorithms were considered in the ensemble model: artificial neural network (ANN; Ripley, 1996), classification tree analysis (CTA; Breiman et al., 1984), flexible discriminant analysis (FDA; Hastie et al., 1994), generalized additive model (GAM; Hastie & Tibshirani, 1990), generalized boosting model (GBM; Ridgeway, 1999), generalized linear model (GLM; McCullagh & Nelder, 1989), multiple adaptive regression splines (MARS; Friedman, 1991), maximum entropy (MAXENT; Phillips et al., 2006), random forest (RF; Breiman, 2001), and surface range envelope (SRE; Busby, 1991). For algorithms requiring species absence records, we generated 10,000 pseudo‐absence points with the equal number as the occupied grids for GAM, GBM, GLM, and RF and 10,000 background points for MAXENT by randomly sampling without replacement. To avoid model over‐fitting, the selection of pseudo‐absence or background points was limited to biological relevance combinations occupied by the species' range map.

To evaluate the accuracy of each algorithm, we performed cross‐validation on each algorithm using bootstrap approach, where random subsets of 80% of each dataset for training data and the remaining 20% for testing algorithm performance using the area under the receiver operating characteristics curve (AUC) and true skill statistics (TSS). This procedure was repeated 10 times to create predictions independent of the training data. Algorithms with AUC >0.90 and TSS >0.80 were considered to have good predictive performance (Allouche et al., 2006; Gallien et al., 2012; Swets, 1988) and were thus kept in the final ensemble model. Based on the selected algorithms in the final ensemble model, we evaluated the relative importance of predictor variables in determining the geographical distribution of *D. involucrate* by calculating the change in correlation between the covariates and the response with and without the selected variable (Thuiller et al., 2015). The final ensemble model was then projected to current and future climatic conditions by using all occurrence and pseudo‐absence data. Finally, these habitat suitability maps were converted to binary presence absence maps using a threshold that maximums model sensitivity plus specificity, which has been shown generally to perform well (Lawson et al., 2014; Liu et al., 2013; Thuiller et al., 2015).

### 2.4 Statistical analysis

Analyses were conducted on the ensemble model map projections of binary presence absence maps. Firstly, to assess potential impacts of climate change on species ranges, following Zhang et al. (2015), we used two metrics to quantify species' vulnerability: the relative change in total area of suitable habitat (CSH) and the loss of current suitable habitat (LSH). The first metric assumes unlimited dispersal into the projected entire suitable habitats in the future time periods and can be calculated using the following equation:

$$\text{CSH}=\left({\text{AREA}}_{\text{future}}‐{\text{AREA}}_{\text{current}}\right)/{\text{AREA}}_{\text{current}}\times 100,$$

where ${\mathrm{AREA}}_{\mathrm{future}}$ and ${\mathrm{AREA}}_{\mathrm{current}}$ are the area of current and future suitable habitats. The second metric assumes no dispersal into the projected suitable habitats out of the current suitable habitats and can be calculated using the following equation:

$$\text{LSH}=\left(1‐\frac{\text{Overlap}\left({\text{AREA}}_{\text{future}}{\text{, AREA}}_{\text{current}}\right)}{{\text{AREA}}_{\text{current}}}\right)\times 100.$$

Secondly, to detect the direction and distance of species range shifts under future conditions, we determined the centroids of current and future binary presence absence maps using the R package “rgeos” with the “gCentroid” function.

Finally, to explore the conservation effectiveness of current nature reserve networks in protecting *D. involucrate* under climate change, we also calculated the area of the current and projected suitable habitat overlapped with the current nature reserve networks, respectively.

## 3 RESULTS

### 3.1 Model performance and variable contribution

The AUC and TSS measures provided highly consistent estimates of the model performance of the 10 modeling algorithms (Table 1). As the mean AUC and TSS values of the 10 modeling algorithms except SRE are all above 0.9 and 0.8, respectively, we removed SRE from the final ensemble model. The AUC and TSS value of the final ensemble model is 0.975 and 0.898, respectively, which is higher than that of the individual modeling algorithms. Among the six selected predictor variables, the temperature annual range is the most influential variable, followed by annual mean temperature, precipitation of the driest month, isothermality, precipitation seasonality, and precipitation of the warmest quarter (Table 2). The response curves of the above six selected predictor variables indicate that *D. involucrate* occurs mainly in areas with annual mean temperature ranging from approximately −0.7 to 19.5℃, isothermality ranging from approximately 6.9–38.2, temperature annual range ranging from approximately 21.9–31.9℃, precipitation of the driest month between about 0.1 and 58 mm, precipitation seasonality between about 39 and 134 mm, and precipitation of the warmest quarter between about 106 and 2,356 mm (Appendices S3–S11).

**TABLE 1:** Performance of 10 modeling algorithms used to predict habitat suitability of *Davidia involucrate*

Modeling algorithms	AUC	TSS
Artificial neural network^a^	0.944 ± 0.006	0.841 ± 0.020
Classification tree analysis^a^	0.926 ± 0.015	0.840 ± 0.026
Flexible discriminant analysis^a^	0.945 ± 0.005	0.822 ± 0.017
Generalized additive model^a^	0.958 ± 0.005	0.858 ± 0.016
Generalized boosting model^a^	0.961 ± 0.007	0.849 ± 0.021
Generalized linear model^a^	0.952 ± 0.003	0.847 ± 0.013
Multiple adaptive regression splines^a^	0.954 ± 0.006	0.846 ± 0.012
MAXENT. Phillips^a^	0.964 ± 0.005	0.851 ± 0.020
Random forest^a^	0.966 ± 0.005	0.870 ± 0.016
Surface range envelope	0.849 ± 0.011	0.697 ± 0.023

Note

Results are shown as mean ± *SE*.

Abbreviations: AUC, area under the receiver operating characteristic curve; TSS, true skill statistics.

^a^ Models were selected to develop the ensemble model.

**TABLE 2:** Relative contributions of the nine selected predictor variables in the ensemble model of habitat suitability for *Davidia involucrate* Ball

Predictor variables	Relative importance
Annual mean temperature (BIO1)	0.344 ± 0.009
Isothermality (BIO3)	0.091 ± 0.003
Temperature annual range (BIO7)	0.834 ± 0.017
Precipitation of the driest month (BIO14)	0.138 ± 0.002
Precipitation seasonality (BIO15)	0.013 ± 0.001
Precipitation of the warmest quarter (BIO18)	0.006 ± 0.001

### 3.2 Species' range shifts under future climatic conditions

**FIGURE 2:**
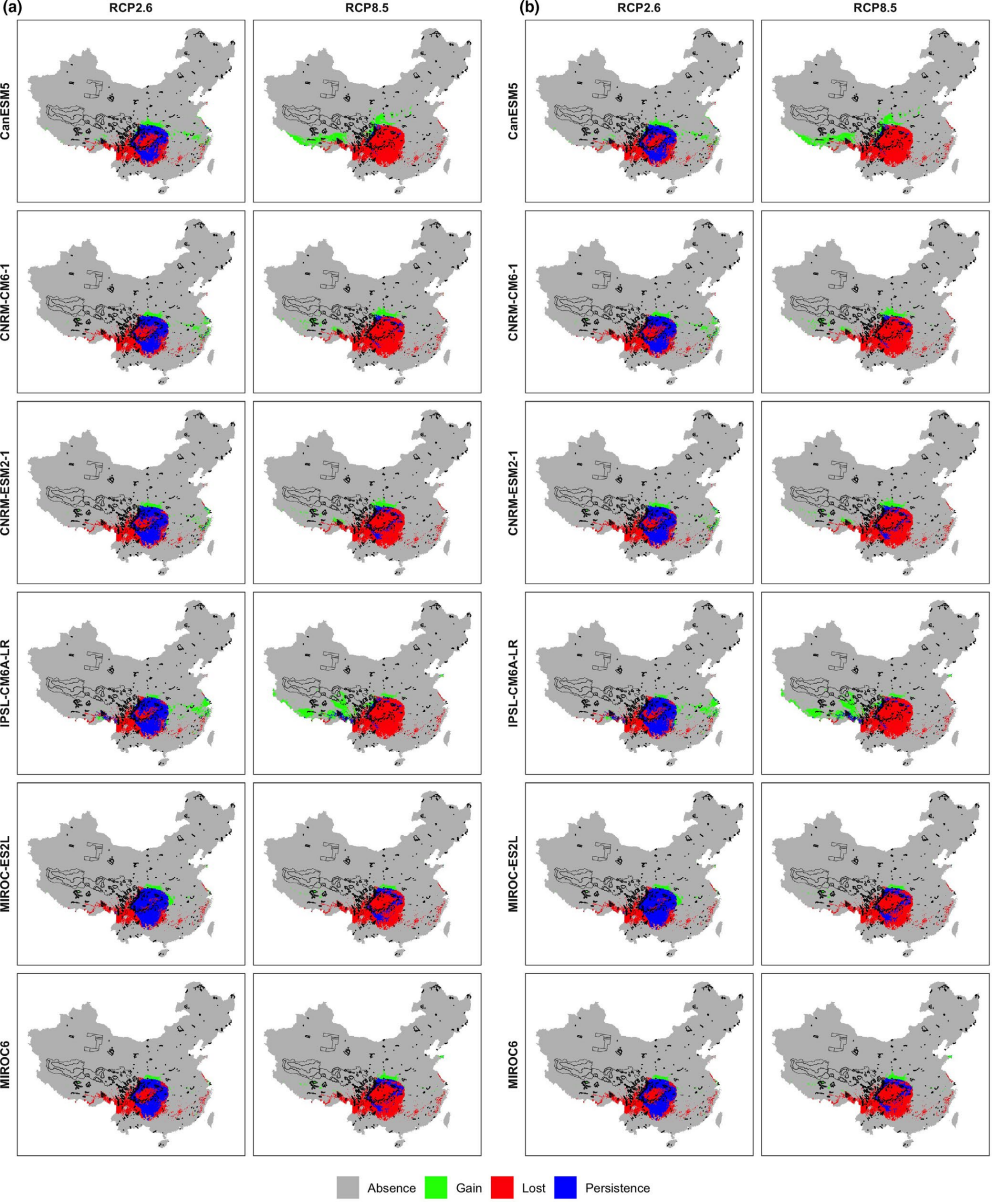
Changes in suitable ranges of *Davidia involucrata* Baill. projected by ensemble SMDs under each GCMs and RCP scenario in: (a) 2050s and (b) 2070s. Four trajectories were assigned to each grid cell by comparing habitat suitability under current and future climatic conditions: “absence,” a grid that is unsuitable for this species under current climatic conditions remain unsuitable under future climatic conditions; “gain,” a grid that is unsuitable for this species under current climatic conditions become suitable under future climatic conditions; “lost,” a grid that is suitable for this species under current climatic conditions become unsuitable under future climatic conditions; “persistence,” a grid that is suitable for this species under current climatic conditions remain suitable under future climatic conditions. The black lines are the boundaries of the current nature reserves

**FIGURE 3:**
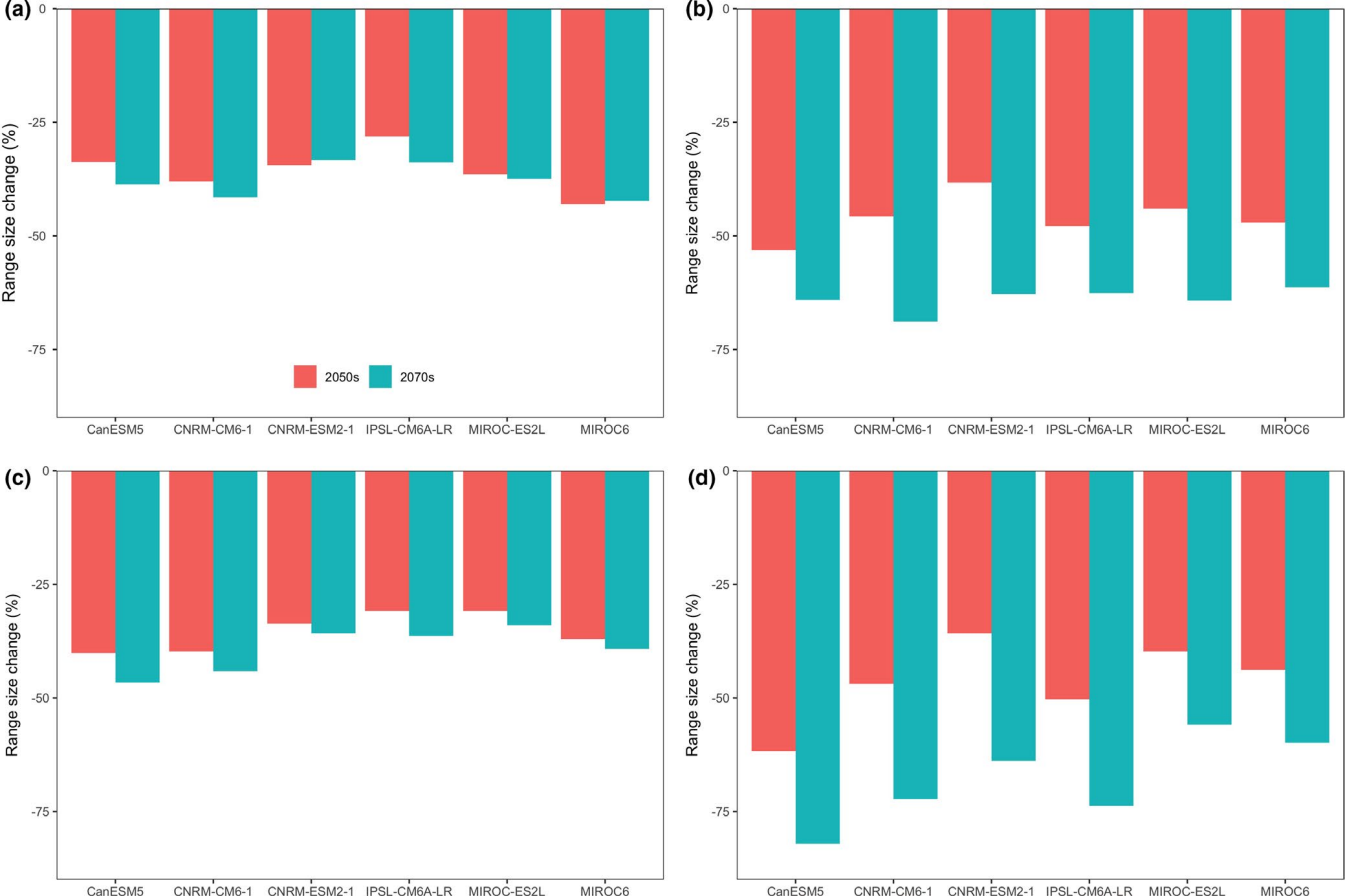
The projected range size changes of *Davidia involucrate* Ball. in 2050s and 2070s. The range size changes (%) are relative to the predicted suitable areas of *D. involucrate* under the current climate conditions. (a)–(d), projections of range size changes of *Davidia involucrate* were obtained from the ensemble models under the unlimited dispersal ((a), (b)) and no dispersal ((c), (d)) scenarios using multiple future climate projections for two time periods from the selected nine GCMs, and under the representative concentration pathway RCP2.6 ((a), (c)) and RCP8.5 ((c), (d)). 48 ensemble models were built for *D. involucrate*. The bars indicate the median value

Under the current conditions, the potential suitable area for *D. involucrate* in China was 959,700 km^2^. Current suitable habitat for *D. involucrate* is mainly distributed in Yunnan, Guizhou, Sichuan, southern Shaanxi, southern Gansu, eastern Tibet, Chongqing, Hubei, and Hunan Provinces (Figure 1). Besides, small areas in Guangxi, Guangdong, Fujian, Jiangxi, Zhejiang, Jiangsu, and Shandong provinces are also predicted to be suitable for *D. involucrate*. The projections of future habitat suitability for *D. involucrate* predicted severe range contraction under all scenarios (Figures 2 and 3). Specifically, by assuming global dispersal, the proportion of current suitable habitats of this species projected to be lost ranged from 28.08% (under IPSL‐CM6A‐LR climate model and RCP 2.6 scenario) to 53.08% (under CanESM5 climate model and RCP 8.5 scenario) by the 2050s, and from 33.27% (under CNRM‐ESM2‐1 climate model and RCP 2.6 scenario) to 68.86% (under CNRM‐CM6‐1 climate model and RCP 8.5 scenario) by the 2070s (Figures 2 and 3a, b). The loss of potential suitable habitats under zero dispersal is more severe than those under global dispersal (Figures 2 and 3c,d), despite having a similar trend in predicted species range size. However, projections indicated that a small part of Gansu, Shaanxi, and Tibet provinces will probably become suitable for *D. involucrate* in the future (Figure 2).

**FIGURE 4:**
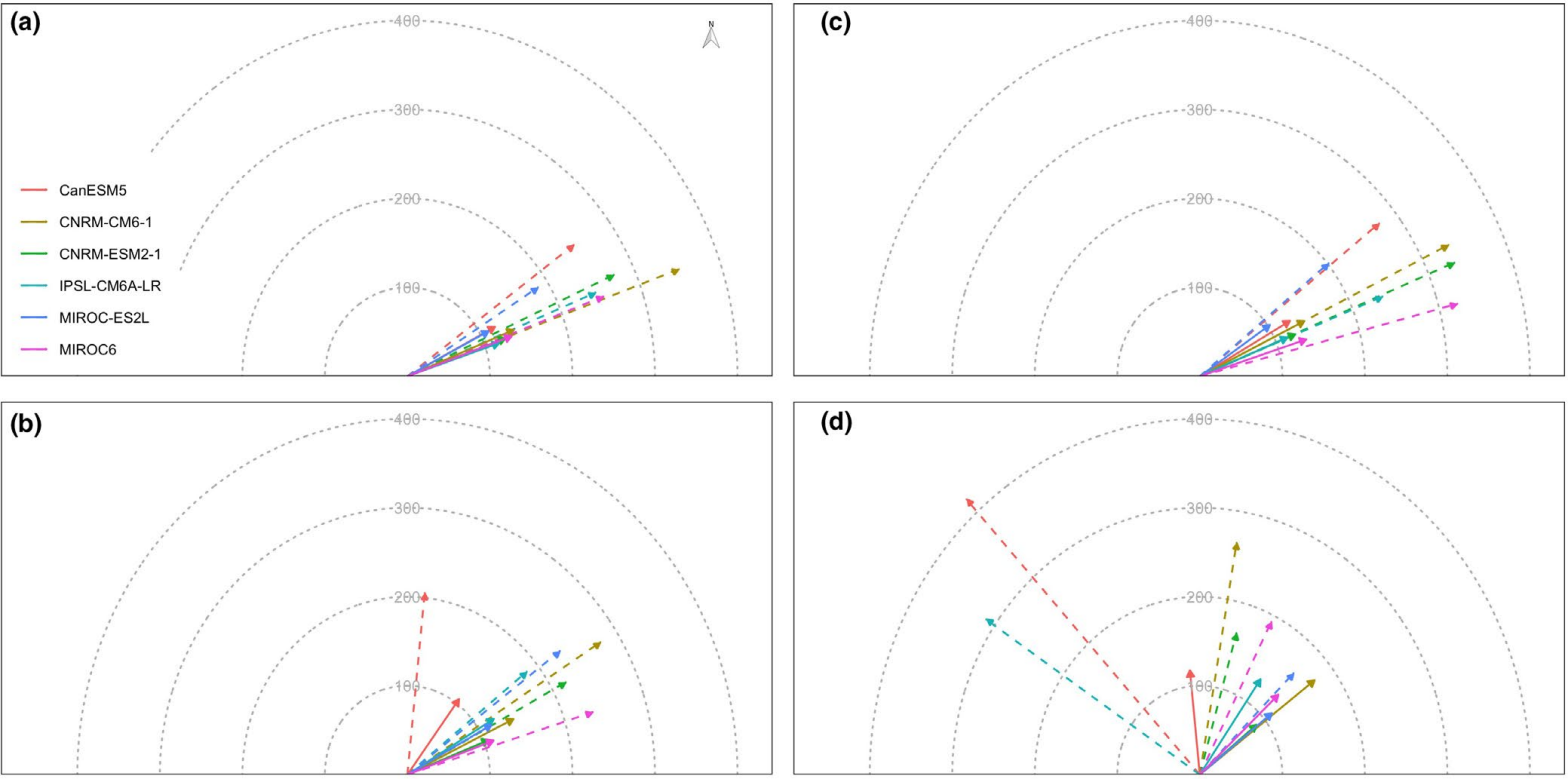
Centroid changes between current distributions of *Davidia involucrate* Ball. and projected distributions by ensemble species distribution model under future changing climate conditions: (a) under RCP 2.6 scenario in 2050s, (b) under RCP 8.5 scenario in 2050s, (c) under RCP 2.6 scenario in 2070s, and (d) under RCP 8.5 scenario in 2070s. The arrow in each map shows direction and distance between present and future distribution centroids. The start of arrow represents centroid of projected suitable area of *D. involucrate* under present climate conditions, while the end coincides with the position of the centroid under future climate scenarios. The real and dashed lines represent global dispersal and no dispersal, respectively

Under future climate conditions, centroids of potential suitable habitats of *D. involucrate* under most scenarios were projected to shift north‐east (Figure 4). The exception is that this species was projected to experience north‐west shifts under CanESM5 and IPSL‐CM6A‐LR climatic models and RCP 8.5 scenario, respectively (Figure 4c,d). The magnitude of species' range shifts varied greatly under different GCMs, different RCPs, and different assumptions of species' dispersal ability (Figure 4). The species would need move from 165.01 km (under the MIROC‐ES2L climate model and RCP 8.5 scenario by the 2070s) to 419.10 km (under the CanESM5 climate model and RCP 8.5 scenario by the 2070s) under global dispersal and from 88.83 km (under the CNRM‐ESM2‐1 climate model and RCP 8.5 scenario by the 2070s) to 175.07 km (under the CNRM‐CM6‐1 climate model and RCP 8.5 scenario by the 2070s) under zero dispersal (Figure 4).

### 3.3 The Effectiveness of current nature reserve networks

**FIGURE 5:**
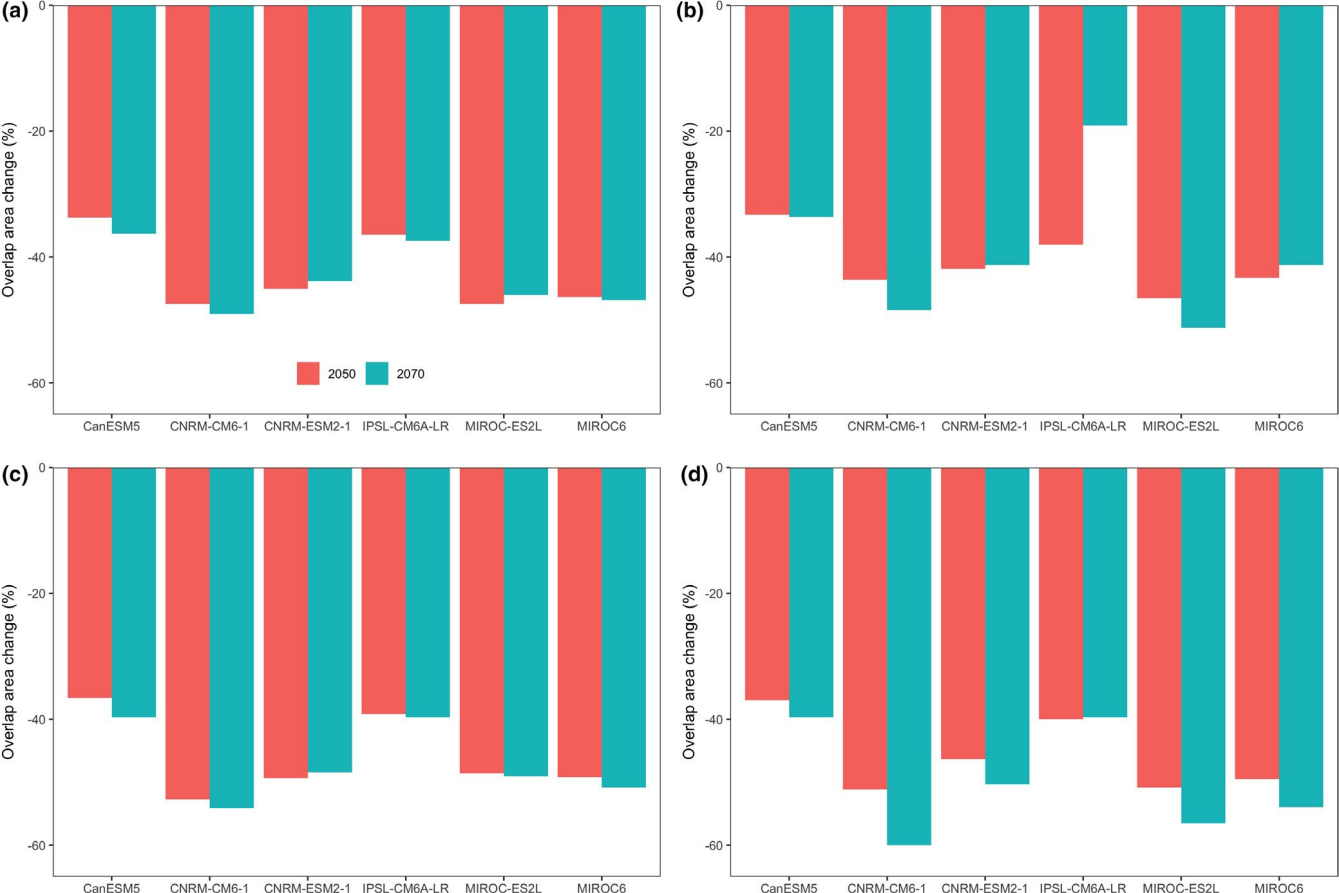
The projected changes of the overlap area of current nature reserve networks and potential suitable habitat of *Davidia involucrate* Ball. in 2050s and 2070s. The overlap area changes (%) are relative to the overlap area of the predicted suitable areas of *D. involucrate* under the current climate conditions and current nature reserve networks. (a)–(d), projections of overlap area changes of *D*. *involucrate* were obtained from the ensemble models under the unlimited dispersal ((a), (b)) and no dispersal ((c), (d)) scenarios using multiple future climate projections for two time periods from the selected nine GCMs, and under the representative concentration pathway RCP2.6 ((a), (c)) and RCP8.5 ((c), (d))

The current nature reserve networks protect 63.18% (628,000 km^2^) of current suitable habitat. However, the overlap area of the current nature reserve networks and projected suitable habitat would decrease severely under all scenarios (Figure 5). Specifically, by assuming global dispersal, the projected changes of the overlap area of the current nature reserve networks and projected suitable habitat ranged from 33.28% (under CanESM5 climate model and RCP 8.5 scenario) to 47.45% (under IPSL‐CM6‐LR climate model and RCP 2.6 scenario) by the 2050s and from 19.11% (under IPSL‐CM6A‐LR climate model and RCP 8.5 scenario) to 57.21% (under MIROC‐ES2L climate model and RCP 8.5 scenario) by the 2070s (Figure 5a,b). Similarly, the overlap area of the current nature reserve networks and projected suitable habitat under zero dispersal is larger than those under global dispersal (Figure 5c,d).

## 4 DISCUSSION

Understanding and predicting how species will response to future climate change is crucial for biodiversity conservation (Wiens et al., 2009) and has required novel approaches with high predictive performance and low predictive uncertainty (Elith et al., 2006; Thuiller et al., 2014). In this study, by using ensemble SDMs and the CMIP6 GCMs, we projected the distribution of suitable habitats of *D. involucrate* under current and future climatic conditions. The results suggest that *D. involucrate* is extremely vulnerability of to future climate change and future climate change would negate the conservation effectiveness of the current nature reserves networks. These findings should inform the dialogue determining where climate change fits into broader picture of conservation for *D. involucrate* and thus have important implications for guiding future conservation planning.

Previous studies have reported that potential suitable habitats of *D. involucrate* were mainly distributed in mountainous areas with narrow annual temperature range and high precipitation (Liu et al., 2019; Su & Zhang, 1999), reflecting this species cold intolerance (Su & Zhang, 1999). Consistent with these previous studies, our results show that, among the selected six climate variables, temperature annual range, annual mean temperature, and precipitation of the driest month were the three most important predictors of the distribution of *D. involucrate*. Therefore, broad temperature annual range, extreme high and/or low temperature, together with low precipitation events in future time periods, could lead to the loss of suitable habitats for this species.

It is often assumed that more complex and more up‐to‐date models will perform better and/or produce more robust projections than previous‐generation models (USGCRP, 2017). Consistent with previous studies (Elith et al., 2006; Thuiller et al., 2014), our results showed that the ensemble SDMs have higher predictive ability than individual SDMs. Furthermore, our future projection suggested that a large proportion of suitable habitats will be lost under future climate change, which has provided useful dialogue informing conservation strategies for *D. involucrate*. Our results revealed that the loss of the suitable habitat of *D. involucrate* would affect the conservation effectiveness of the current nature reserve networks, which does not protect the current suitable habitat of *D. involucrate* adequately, nor will they protect future potential suitable habitat. These findings also inform a critical initial step in implementing the adaptation planning processes for protecting *D. involucrate* (Yu et al., 2017; Zhang et al., 2017). On one hand, some nature reserves located in Sichuan Basin would suffer the greatest loss of suitable habitat under future climate change (Figure 2). These nature reserves are urgent need to be reevaluated under the background of climate change (Bellard et al., 2012; Hansen et al., 2010), and coping strategies to deal with these potential threats require further in‐depth study; on the other hand, a part of Sichuan, Gansu, Shaanxi, and Tibet provinces were identified to be the potential climatic refuges (i.e., unchanged and new gained suitability habitat; Ashcroft, 2010) for *D. involucrate* (Figure 2). It is urgent needs to design these regions as priority for conservation and to establish new nature reserves in the currently unprotected areas (e.g., southern Shaanxi and southern Gansu) in these regions.

Despite the predictive power of their ensemble modeling of *D. involucrate*, an important limitation in the present study is that we assessed future habitat suitability under two extreme dispersal assumptions (i.e., no dispersal and unlimited dispersal), which ignores the realistic rates and modes of dispersal of this species (Saupe et al., 2012). These assumptions are likely inaccurate, which could lead to overestimation of suitable habitat under the unlimited dispersal assumption or underestimation under the zero‐dispersal assumption (Engler & Guisan, 2009; Viana, 2017; Zanatta et al., 2020). For instance, Engler and Guisan (2009) assessed the potential impacts of climate change on habitat suitability of 287 mountain plants under four dispersal scenarios (unlimited dispersal, zero dispersal, realistic dispersal, and realistic dispersal with long‐distance dispersal events). Their result showed that the projected future distributions under realistic dispersal were significantly different from those of other dispersal scenarios. However, regardless of dispersal scenario, our results highlight the high vulnerability of *D. involucrate* to climate change and provide the bounds to the magnitude of the change.

Overall, our research provides fundamental knowledge for understanding the potential impacts of climate change on the distribution of *D. involucrate*. This study also provides useful information for comprehending vegetation changes at global scales under climate change, especially for the climate‐related range shifts of Tertiary relict plants (Tang et al., 2017). However, to effectively improve the predictive power of SDM projections, we recommend incorporating diverse ecological processes, such as morphology and dispersal strategies, into the future projections.

## CONFLICT OF INTEREST

None declared.

## AUTHOR CONTRIBUTIONS

**Teng Long:** Conceptualization (equal); formal analysis (lead); investigation (lead); methodology (equal); project administration (equal); writing–original draft (lead); writing–review and editing (equal). **Junfeng Tang:** Formal analysis (supporting); methodology (equal); supervision (equal); writing–review and editing (equal). **Nicholas Pilfold:** Formal analysis (equal); methodology (equal); writing–review and editing (equal). **Xuzhe Zhao:** Conceptualization (equal); funding acquisition (lead); investigation (equal); project administration (lead); resources (lead); supervision (lead); writing–original draft (equal); writing–review and editing. **Tingfa Dong:** Conceptualization (equal); project administration (equal); supervision (equal); writing–review and editing.

## Supporting information

Appendix S1‐S11

## Data Availability

Datasets used in this study are available online from the Dryad Digital Repository: https://doi.org/10.5061/dryad.cnp5hqc56.
