# Supplementary material for: Predicting range shifts of Davidia involucrata Ball. under future climate change
Source: Ecol Evol. 2021 Aug 11;11(18):12779–89. doi: 10.1002/ece3.8023 (PMC8462142; doi:10.1002/ece3.8023)
Supplement: Supplementary file 1 — Appendix S1‐S11 [file ECE3-11-12779-s001.docx]

**Appendix S1.** Definition and description of climate variables in our datasets.

| Variables | Variable description and description | Units |
| --- | --- | --- |
| BIO1 | Mean annual temperature in each grid cell | ℃ |
| BIO2 | Mean diurnal range (mean of maximal temperature - minimal temperature) in each grid cell | ℃ |
| BIO3 | Isothermality (Bio2/Bio7) (* 100) in each grid cell |  |
| BIO4 | Temperature seasonality (standard deviation *100) in each grid cell | ℃ |
| BIO5 | Maximal temperature of the warmest month in each grid cell | ℃ |
| BIO6 | Minimal temperature of coldest month in each grid cell | ℃ |
| BIO7 | Temperature annual range (Bio5-Bio6) in each grid cell | ℃ |
| BIO8 | Mean temperature of the wettest quarter in each grid cell | ℃ |
| BIO9 | Mean temperature of the driest quarter in each grid cell | ℃ |
| BIO10 | Mean temperature of the warmest quarter in each grid cell | ℃ |
| BIO11 | Mean temperature of the coldest quarter in each grid cell | ℃ |
| BIO12 | Total annual precipitation in each grid cell | mm |
| BIO13 | Precipitation of the wettest month in each grid cell | mm |
| BIO14 | Precipitation of the driest month in each grid cell | mm |
| BIO15 | Precipitation seasonality (coefficient of variation) in each grid cell |  |
| BIO16 | Precipitation of the wettest quarter in each grid cell | mm |
| BIO17 | Precipitation of the driest quarter in each grid cell | mm |
| BIO18 | Precipitation of the warmest quarter in each grid cell | mm |
| BIO19 | Precipitation of the coldest quarter in each grid cell | mm |

**Appendix S2.** The values (right-upper triangle) and corresponding significance (left-lower triangle) of Pearson correlation coefficients between the ten selected variables, examined at three grid sizes and three time periods.

|  | BIO1 | BIO3 | BIO7 | BIO14 | BIO15 | BIO18 |
| --- | --- | --- | --- | --- | --- | --- |
| BIO1 |  | -0.208 | -0.344 | 0.593 | -0.480 | 0.397 |
| BIO3 | *** |  | -0.542 | -0.137 | 0.161 | -0.016 |
| BIO7 | *** | *** |  | -0.510 | 0.388 | -0.593 |
| BIO14 | *** | *** | *** |  | -0.653 | 0.527 |
| BIO15 | *** | *** | *** | *** |  | -0.260 |
| BIO18 | *** | *** | *** | *** | *** |  |

*** P<0.001; ** P<0.01; * P<0.05; ns, not significant.


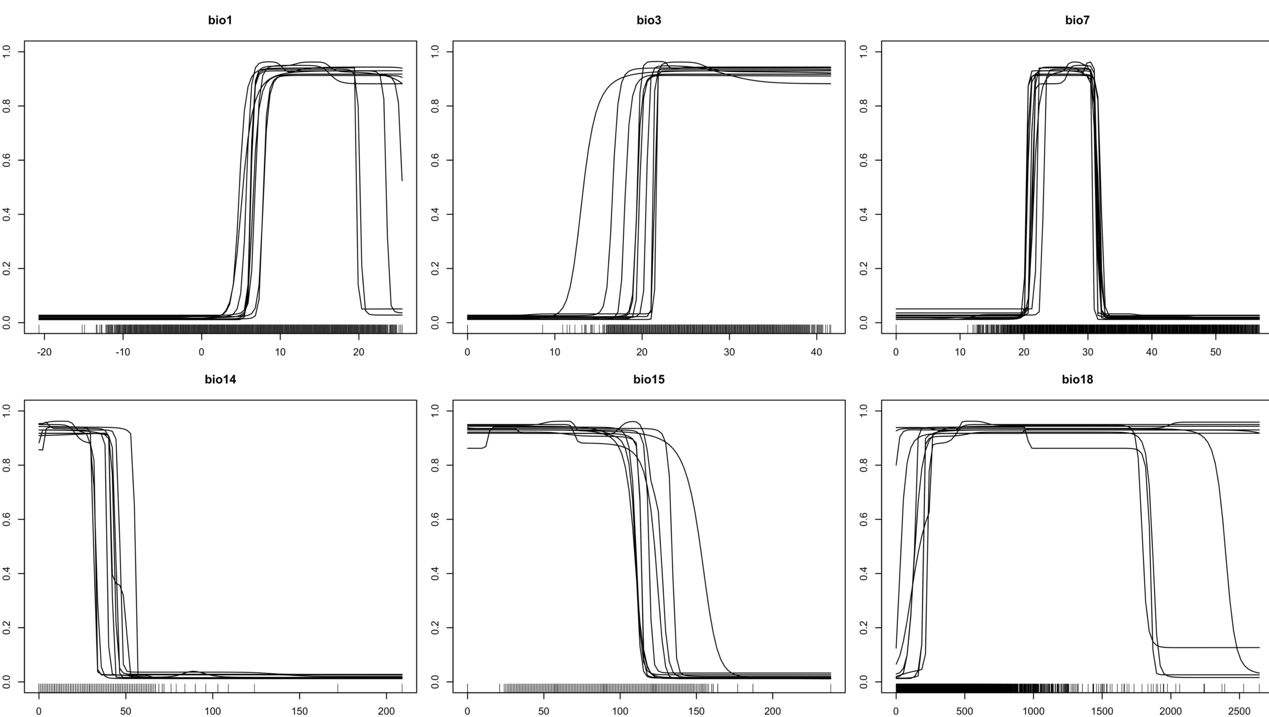


**Appendix S3** Response curves of annual mean temperature (bio1), isothermality (bio3), temperature annual range (bio7), precipitation of the driest month (bio14), precipitation seasonality (bio15) and precipitation of the warmest quarter (bio18) in artificial neural network (ANN) models for *D. involucrate*.


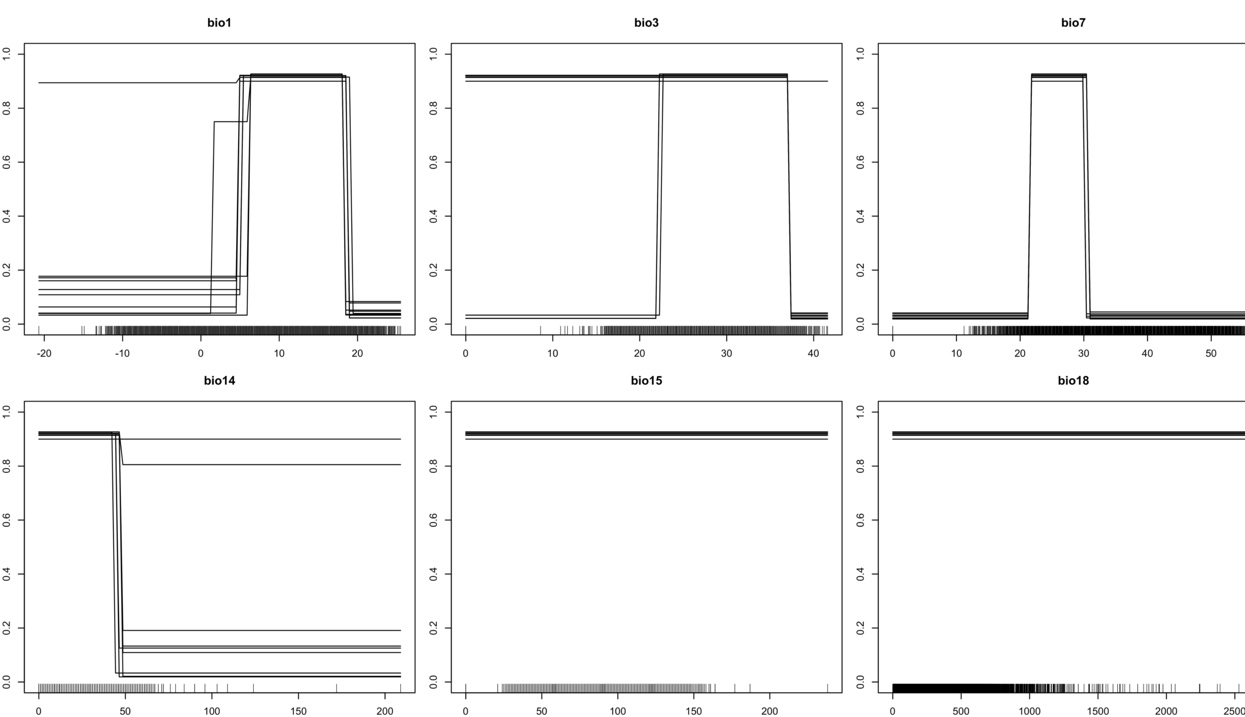


**Appendix S4** Response curves of annual mean temperature (bio1), isothermality (bio3), temperature annual range (bio7), precipitation of the driest month (bio14), precipitation seasonality (bio15) and precipitation of the warmest quarter (bio18) in Classification tree analysis (CTA) models for *D. involucrate*.


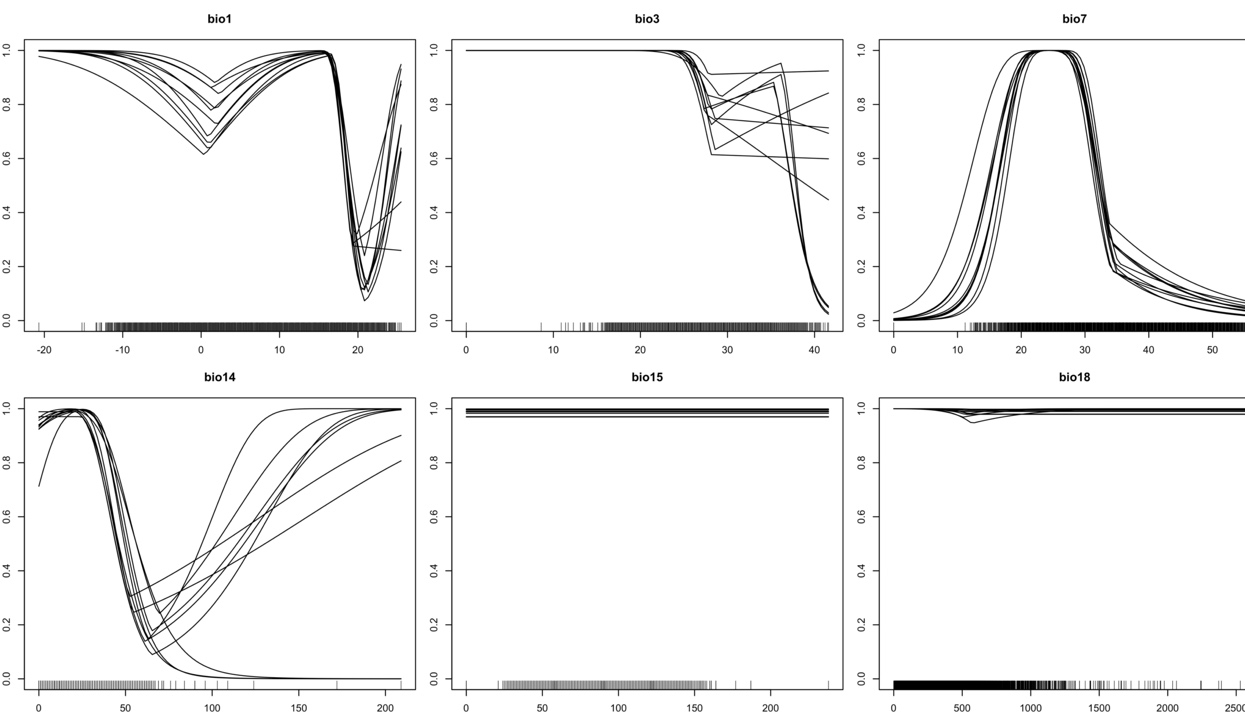


**Appendix S5** Response curves of annual mean temperature (bio1), isothermality (bio3), temperature annual range (bio7), precipitation of the driest month (bio14), precipitation seasonality (bio15) and precipitation of the warmest quarter (bio18) in flexible discriminant analysis (FDA) models for *D. involucrate*.


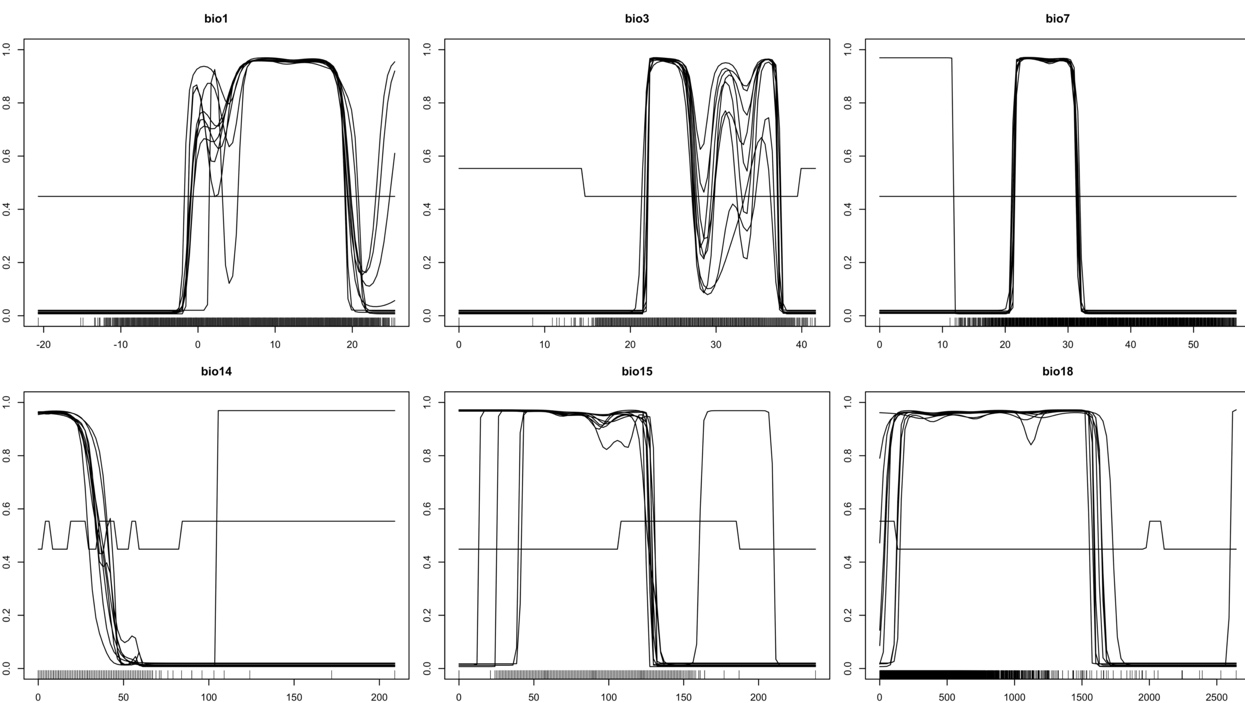


**Appendix S6** Response curves of annual mean temperature (bio1), isothermality (bio3), temperature annual range (bio7), precipitation of the driest month (bio14), precipitation seasonality (bio15) and precipitation of the warmest quarter (bio18) in generalised additive model (GAM) models for *D. involucrate*.


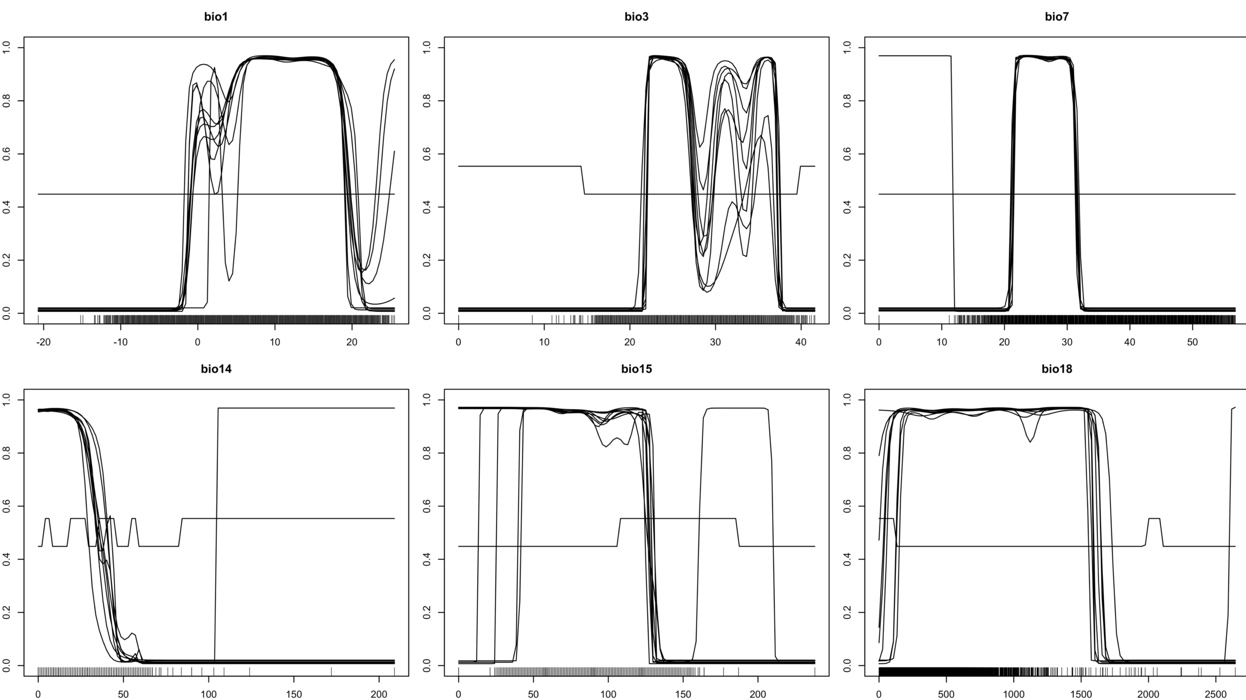


**Appendix S7** Response curves of annual mean temperature (bio1), isothermality (bio3), temperature annual range (bio7), precipitation of the driest month (bio14), precipitation seasonality (bio15) and precipitation of the warmest quarter (bio18) in generalised boosting model (GBM) models for *D. involucrate*.


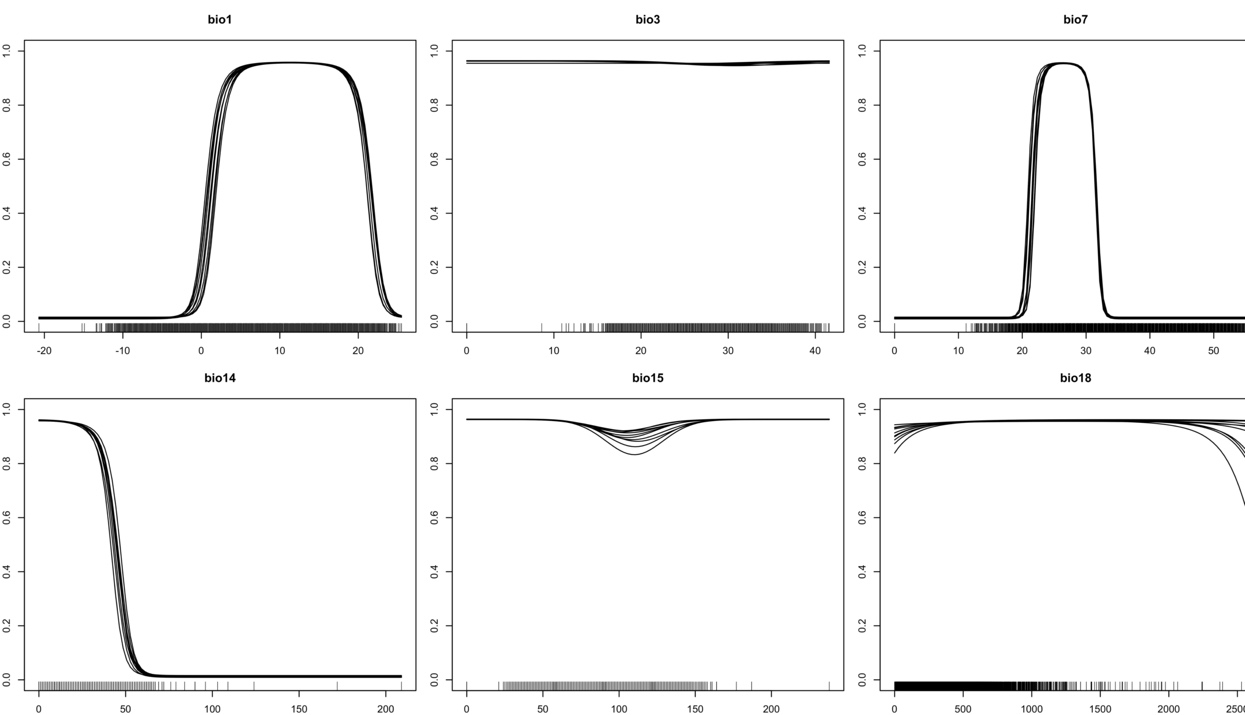


**Appendix S8** Response curves of annual mean temperature (bio1), isothermality (bio3), temperature annual range (bio7), precipitation of the driest month (bio14), precipitation seasonality (bio15) and precipitation of the warmest quarter (bio18) in generalised linear model (GLM) models for *D. involucrate*.


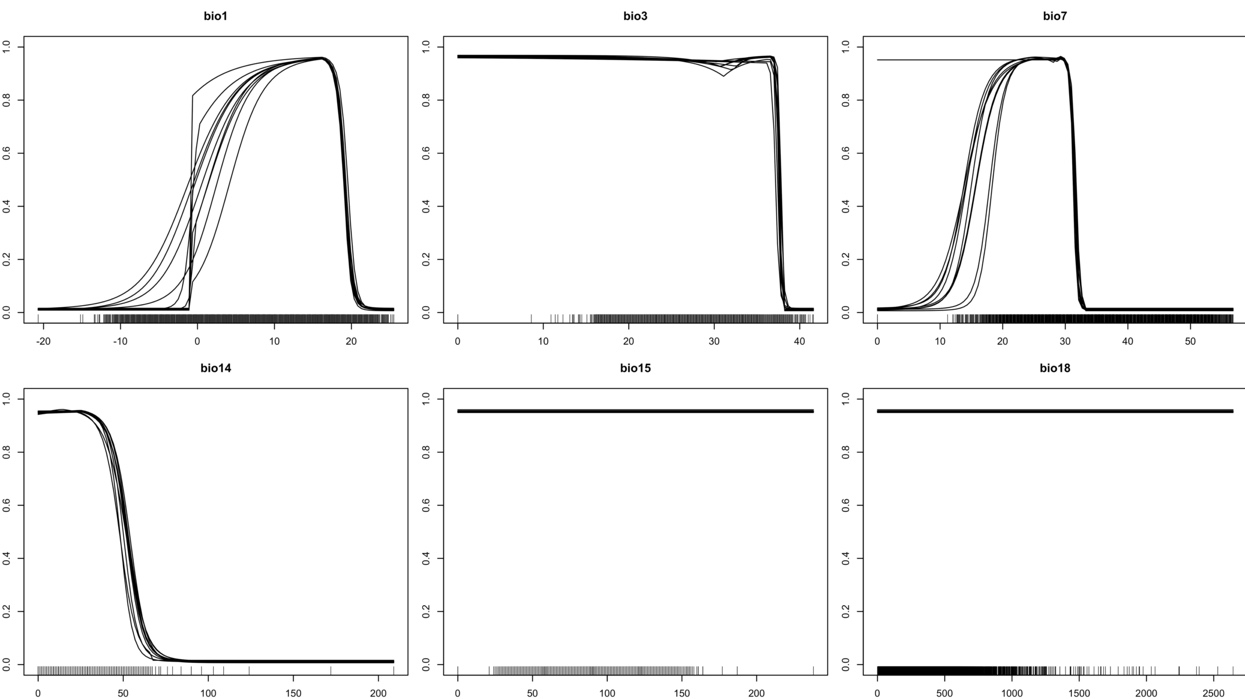


**Appendix S9** Response curves of annual mean temperature (bio1), isothermality (bio3), temperature annual range (bio7), precipitation of the driest month (bio14), precipitation seasonality (bio15) and precipitation of the warmest quarter (bio18) in multiple adaptive regression splines (MARS) models for *D. involucrate*.


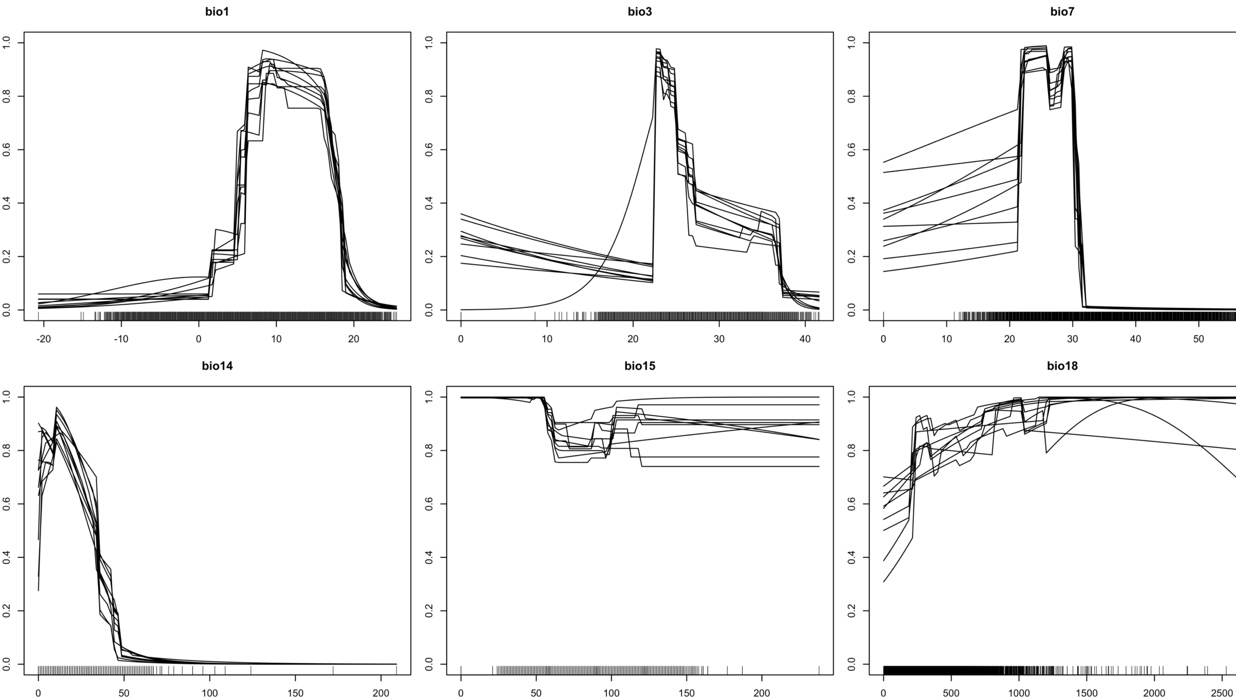


**Appendix S10** Response curves of annual mean temperature (bio1), isothermality (bio3), temperature annual range (bio7), precipitation of the driest month (bio14), precipitation seasonality (bio15) and precipitation of the warmest quarter (bio18) in Maxent models for *D. involucrate*.


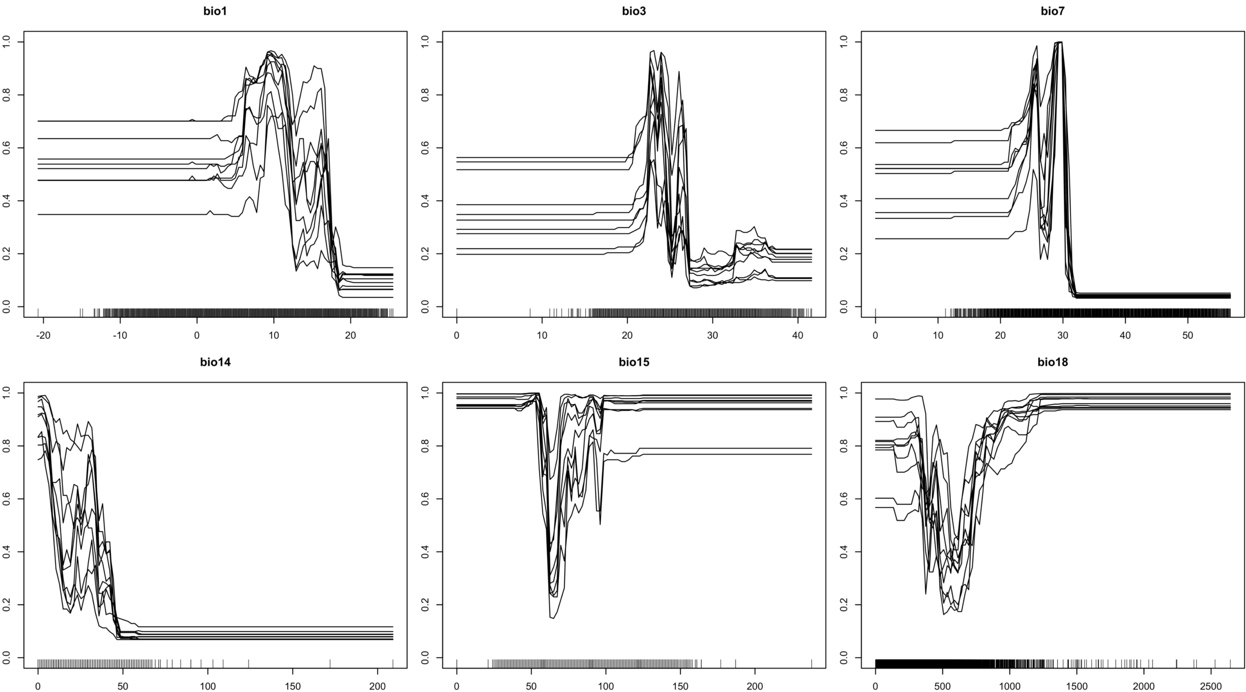


**Appendix S11** Response curves of annual mean temperature (bio1), isothermality (bio3), temperature annual range (bio7), precipitation of the driest month (bio14), precipitation seasonality (bio15) and precipitation of the warmest quarter (bio18) in random forest (RF) models for *D. involucrate*.
